# Deep-learning computer-aided detection and classification of prostate lesions on biparametric MRI: comparison with expert readers

**DOI:** 10.1186/s13244-026-02261-0

**Published:** 2026-04-22

**Authors:** Deepak Jain, Francisco Restrepo, Kazuya Yasokawa, Efe Ozkaya, Mickael Tordjman, Marc Berns, Alon Slutzky, Julian Franko, Octavia Bane, Heinrich von Busch, Robert Grimm, Ashutosh K. Tewari, Sara Lewis, Bachir Taouli

**Affiliations:** 1 Department of Diagnostic, Molecular and Interventional Radiology, Icahn School of Medicine at Mount Sinai, New York, NY USA; 2 Biomedical Engineering and Imaging Institute, Icahn School of Medicine at Mount Sinai, New York, NY USA; 3 Department of Diagnostic Radiology, Kawasaki Medical School, Kurashiki, Japan; 4 Siemens Healthineers AG, Erlangen, Germany; 5 Department of Urology, Icahn School of Medicine at Mount Sinai, New York, NY USA

**Keywords:** Prostate cancer, Magnetic resonance imaging, Deep learning, Diagnosis (computer-assisted)

## Abstract

**Objective:**

To assess the performance of a deep learning-based computer-aided detection (DL-CAD) algorithm for prostate lesion detection and classification on biparametric (bp)MRI.

**Materials and methods:**

This retrospective, single-center study included men undergoing 3-T MRI of the prostate for suspected prostate cancer (PCa) between July and September of 2022. Using the radiology report as the reference standard, detection performance for high-risk lesions (defined as PI-RADS ≥ 3, 4, 5) by the DL-CAD was evaluated per-patient using sensitivity, specificity, PPV, NPV and AUC; and per-lesion using sensitivity and PPV. Kappa statistics was used to assess per-patient detection and per-lesion classification of PI-RADS ≥ 3 lesions. Clinical and imaging factors associated with discordance between DL-CAD and radiology reports were assessed using Mann–Whitney, Chi-square, and Fisher’s exact tests.

**Results:**

442 adult males (mean age 65 ± 9 years) were assessed. Per-patient sensitivity, specificity, PPV, and NPV for detection of PI-RADS ≥ 4 and 5 lesions were 65.3%/81.2%/62.7%/82.9% and 82.1%/93.8%/65.7%/97.3%, respectively. Per-patient performance for identifying PI-RADS ≥ 3/4/5 lesions was fair-to-excellent: AUC = 0.67 (0.62–0.71)/0.75 (0.71–0.80)/0.92 (0.89–0.96). For detection of PI-RADS ≥ 4 and 5, per-lesion sensitivity was 60.4% and 78.3%, while PPV was 55.0% and 60.3%. Per-patient agreement between DL-CAD and the reference increased with higher PI-RADS scores (kappa = 0.26 (0.18–0.35)/0.46 (0.37–0.55)/0.68 (0.59–0.78)). Agreement on classification of PI-RADS ≥ 3 lesions was moderate (kappa = 0.56 (0.45–0.68)).

**Conclusion:**

A pre-trained DL-CAD showed good-to-excellent per-patient performance for the detection of PI-RADS ≥ 4 lesions and moderate performance of PI-RADS ≥ 3 lesion classification. Future prospective studies validating the DL algorithm with histopathologic correlation are warranted.

**Critical relevance statement:**

A deep learning computer-aided detection (DL-CAD) algorithm showed good-to-excellent per-patient performance for detection of PI-RADS ≥ 4 lesions, moderate performance of PI-RADS ≥ 3 lesion classification and high negative predictive value, which can be applied in the clinic with knowledge of its limitations.

**Key Points:**

- Clinical validation of deep learning computer-aided detection (DL-CAD) models for the detection and classification of prostate lesions on MRI is urgently needed.
- A pre-trained DL-CAD algorithm showed fair-to-excellent per-patient performance for detection of prostate lesions on biparametric MRI, with moderate performance for PI-RADS ≥ 3 lesion classification.
- Identification of false negatives and false positives of prostate cancer detection DL-CAD algorithms is important for future improvement and clinical deployment.
- A DL-CAD-based prostate cancer detection algorithm with high NPV may reduce interpretation time.

**Graphical Abstract:**

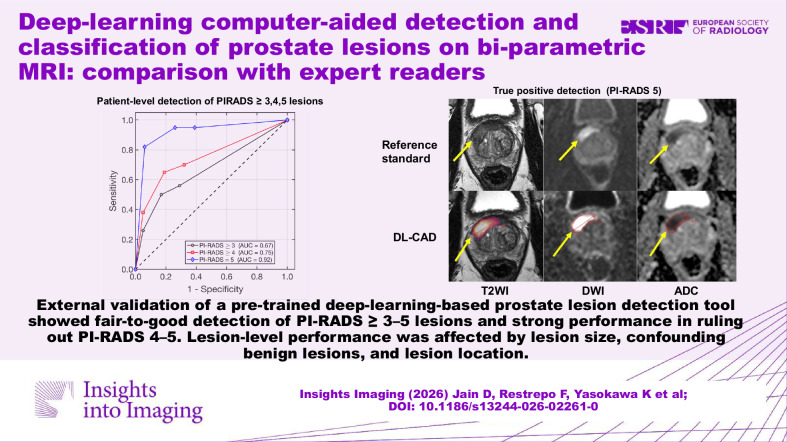

## Introduction

Prostate MRI plays a central role in the care of men with suspected prostate cancer (PCa), including roles in diagnosis, staging, risk stratification, and post-treatment assessment, with high diagnostic accuracy for the detection of clinically significant PCa [1]. However, despite improvements in standardization of MR image interpretation including the development of the Prostate Imaging-Reporting and Data System (PI-RADS) [2, 3], clinical interpretation of prostate MRI still suffers from significant intra- and inter-reader variability [4, 5], in both multiparametric (mp)MRI and the shorter biparametric (bp)MRI protocol, consisting of T2-weighted (T2WI) and diffusion-weighted (DWI) imaging [6, 7].

Deep Learning-based computer-aided detection (DL-CAD) algorithms for detection and classification of prostate lesions may improve radiologists’ diagnostic accuracy while reducing inter-reader variability and interpretation time [8, 9]. As standalone tools, some DL-CAD algorithms have shown comparable lesion detection performance to radiological assessment [10, 11] and reliable prediction of clinically significant PCa at histopathologic examination [12–14]. Previous studies also demonstrated increased sensitivity, accuracy, and inter-reader agreement in the detection of prostate lesions on bpMRI [15] and mpMRI [16, 17] when radiologists’ assessments were complemented with CAD predictions.

Despite these advances, there remains an unmet need for CAD algorithms that not only identify patients at risk based on a single index lesion but that can also correctly locate, segment, and classify all suspicious prostate lesions visible on MRI. This is especially relevant for correct radiation targeting/dosage and targeted prostate biopsies [18, 19]. On the other hand, CAD algorithms that can reliably rule out clinically significant PCa and avoid unnecessary biopsies are also valuable in clinical practice [20].

The purpose of our study was to assess the performance of a pre-trained DL-CAD algorithm for prostate lesion detection and classification on bpMRI.

## Materials and methods

### Patient cohort

**Fig. 1:**
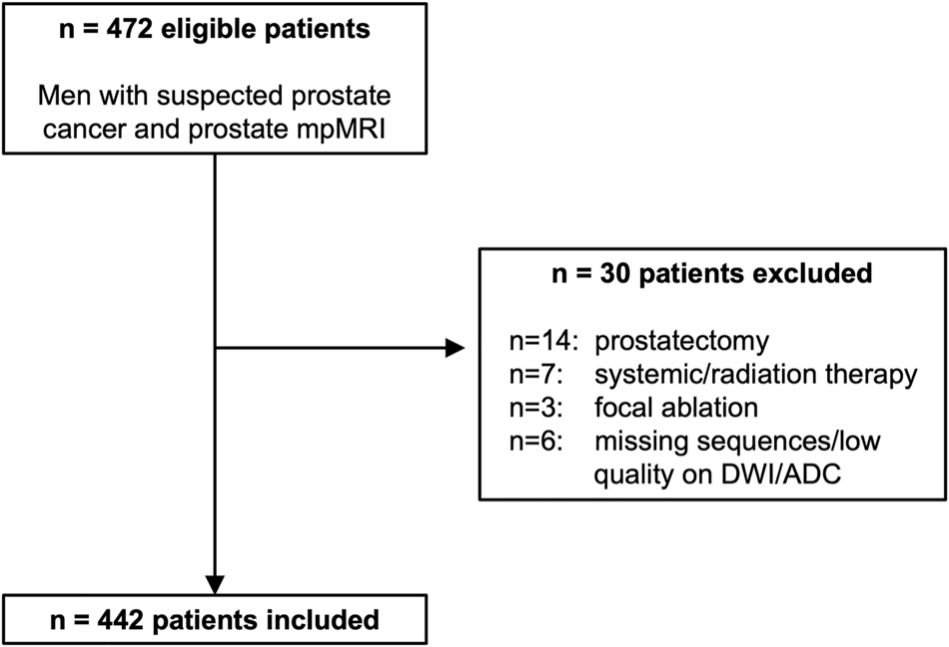
Flowchart of study cohort. ADC, apparent diffusion coefficient; DWI, diffusion-weighted imaging; mpMRI, multiparametric MRI

This single-center retrospective study was approved by the Icahn School of Medicine at Mount Sinai’s Program for the Protection of Human Subjects (reference number: Study-21-01490), with a waiver for informed consent. The imaging database was queried from July 1st, 2022, to September 30th, 2022, for consecutive men over 18 years of age who underwent prostate MRI on a 3-T system for the indication of clinically suspected PCa. Exclusion criteria are listed in the study flow-chart (Fig. 1). Finally, 442 patients were eligible for the study. Table 1 summarizes the patient characteristics for the study cohort.

**Table 1:** Cohort characteristics (*n* = 442)

Clinical variable	Mean (SD)
Age (years)	65.1 (8.7)
BMI (kg/m^2^)^a^	27.2 (4.1)
Prostate-specific antigen (ng/mL)^b^	8.4 (16.9)
Prostate-specific antigen density (ng/mL^2^)^b^	0.2 (0.3)
MRI prostate volume (mL)	67.1 (46.7)

PI-RADS score distribution (reference standard)	Number of lesions (%)
Total number of lesions	294
PI-RADS 1–2	20 (6.8)
PI-RADS 3	92 (31.3)
PI-RADS 4	122 (41.5)
PI-RADS 5	60 (20.4)

Index lesion location (reference standard)	Number of lesions (%)
Total index lesions	224
TZ	76 (34)
PZ	144 (64)
TZ/PZ	4 (2)

T2WI/DWI image quality scores	Number of patients (%) T2WI/DWI
1 (nondiagnostic)	1 (0%)/1 (0%)
2 (poor)	1 (0%)/3 (1%)
3 (satisfactory)	18 (4%)/30 (7%)
4 (good)	139 (32%)/129 (29%)
5 (excellent)	283 (64%)/279 (63%)

Scanner	Number of patients (%)
Magnetom Skyra, Siemens Healthineers	381 (86)
Discovery MR 750, GE Medical Systems	61 (14)

*BMI* body-mass index, *DWI* diffusion-weighted imaging, *PZ* peripheral zone, *T2WI* T2-weighted imaging, *TZ* transition zone

^a^ BMI not available in 31 subjects

^b^ PSA not available in 15 subjects

### MRI protocol

All patients underwent MRI of the prostate using one of two clinical 3-T MRI systems (MAGNETOM Skyra, Siemens Healthineers; or GE Premier, GE Healthcare) as per the clinical protocol at our institution. The MRI protocol included axial, coronal, and sagittal FSE-T2WI, axial DWI (b-values: 50, 1000, 1600) and ADC maps computed with b = 50, 1600. The MRI protocol remained stable during the study period.

### Data collection and qualitative analysis

Data was extracted by the study coordinator (Observer 1, D.J., an MRI fellow with 1 year of experience) from the clinical MRI report, including prostate volume, prostate lesion location (when present, peripheral zone (PZ)/transition zone (TZ); right/left; base/midgland/apex), maximum lesion size on axial imaging (mm), and biparametric PI-RADS v2.1 scores for each lesion. Overall image quality (PI-QUAL) scores [21] tailored for bpMRI were evaluated on axial T2WI and DWI/ADC by Observer 1 using a 5-point scale (1: nondiagnostic; 2: poor image quality; 3: satisfactory image quality; 4: good image quality; 5: excellent image quality), considering image sharpness, motion and distortion together.

### DL algorithm and image analysis

**Fig. 2:**
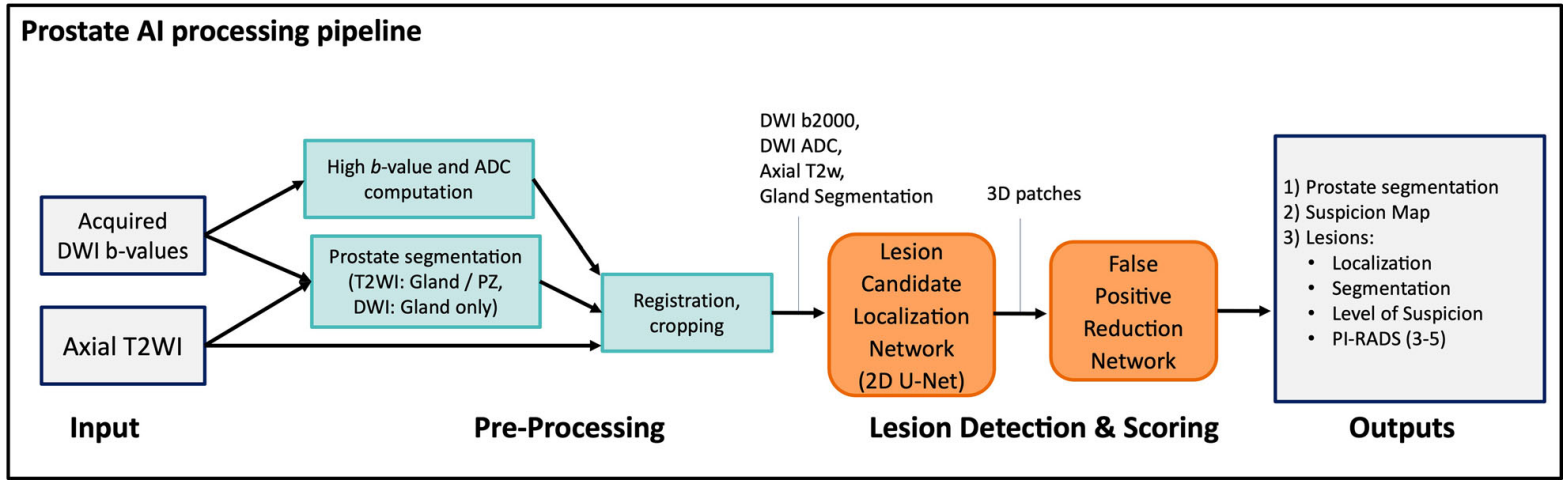
Overview of the deep learning-based computer-aided detection (DL-CAD) algorithm (Siemens Healthineers). Whole-gland segmentation is first performed on T2WI volumes and b2000 DWI and ADC images (using b = 50, 1000) are calculated. Rigid registration is applied to align DWI to T2WI images, which are then fed into a 2D UNet-based CNN for segmentation of initial lesion candidates. 3D patches containing these lesions are cropped and analyzed by a subsequent false-positive reduction stage to finally produce an AI level of suspicion (LoS) for each lesion in the range 1–100 and a PI-RADS score (3–5). CNN, convolutional neural network; DWI, diffusion-weighted imaging; PZ, peripheral zone; T2WI, T2-weighted imaging; TZ, transitional zone

A proprietary DL-CAD system (MR-Prostate AI research application v1.3.4; Siemens Healthineers) consisting of a DL-based lesion detection and classification algorithm pre-trained with 3087 bpMRIs from 7 European and Asian institutions [22] was used in this study. This was obtained through a research collaboration agreement with Siemens Healthineers. An overview of the DL-CAD pipeline is depicted in Fig. 2 and is briefly described as follows. First, whole-gland segmentation is performed on T2WI volumes using a DL-based method reported previously [23]. The software internally calculates b2000 DWI and ADC images (using b = 50, 1000) from the acquired DWI series. Rigid registration is applied to align DWI to T2WI images [24], which are then fed into a 2D UNet-based convolutional neural network, trained on PI-RADS ≥ 3 lesions (based on expert radiologists’ interpretation), to detect initial lesion candidates. This lesion detection module was previously trained [23] using an encoder-decoder architecture with 16 blocks, kernel size 3 × 3, batch normalization, and a Leaky ReLU activation. Multi-slice patches around these initial candidates were cropped and analyzed by a subsequent false-positive reduction stage trained using lesions with corresponding biopsy as ground truth, producing an AI level of suspicion (LoS) for each candidate in the range of 1–100. This false-positive reduction module was trained previously [22] using the ADAM [25] optimizer for 100 epochs, with L2 regularization of 10^−^^4^, data augmentation ([−45° to 45°]-rotations, [−5 to 5]-pixel translations, and vertical flips), and a weighted binary cross-entropy loss function. Lesion candidates with an LoS of less than 60, corresponding to an estimated PI-RADS 1–2 score, are discarded, while an AI-based PI-RADS 3 score is assigned for lesion detections with an LoS of 60–79. For detections with an LoS of 80–100, a PI-RADS 4 score is assigned if the segmentation diameter is less than 15 mm, and a PI-RADS 5 score otherwise. Overall, the DL-CAD algorithm output includes whole prostate and lesion segmentations, as well as a report containing size, location, and estimated PI-RADS score for every detected prostate lesion.

The acquired T2WI and DWI DICOM files from all patients were extracted from the local PACS system and analyzed by the DL-CAD on an AMD Ryzen 7-2700X Eight-Core Processor. All patient data from our cohort were used as an external test set, and re-training was not performed.

### Reference standard

Clinical MRI reports from 7 fellowship-trained radiologists with experience ranging from 5 to 20 years were taken as the reference standard. Clinical MRI interpretation based on expert radiological assessment using PI-RADSv2.1 provides a relevant benchmark, representing the current clinical practice standard, especially given that the pathologic correlation was not available in all of our study population.

Data extracted from the clinical reports included lesion location, maximum axial diameter (mm), and PI-RADS scores for both T2WI and DWI sequences. The biparametric (bp)PI-RADS score was obtained retrospectively from the clinical report by Observer 1 as per PI-RADSv2.1. This score was chosen for the statistical analysis. PI-RADS scores assigned by the reference standard and DL-CAD were binarized with increasing cutoffs (PI-RADS = 3, 4, 5) to effectively distinguish between high- and low-risk scores. For each PI-RADS cutoff, contingency tables for the per-patient and per-lesion analyses were generated reflecting the frequencies of true positive (TP), true negative (TN), false positive (FP), and false negative (FN) detections.

Per-patient detections were defined as follows: (1) TP: defined as at least one PI-RADS ≥ 3–5 lesion detected at the same location by both the DL-CAD and the reference standard (regardless of PI-RADS 3–5 score), (2) FP: defined as at least one PI-RADS ≥ 3–5 lesion detected by the DL-CAD and no such lesion detected by the reference standard, (3) FN: defined as at least one PI-RADS ≥ 3–5 lesion detected by the reference standard and not detected by the DL-CAD, (4) TN: defined as no PI-RADS ≥ 3–5 lesions detected by either DL-CAD or the reference standard.

Per-lesion detections were defined as follows: (1) TP: defined as all PI-RADS ≥ 3–5 lesions detected at the same location by both the DL-CAD and the reference standard, (2) FP: defined as PI-RADS ≥ 3–5 lesions detected by the DL-CAD and no such lesion detected by the reference standard, (3) FN: defined as PI-RADS ≥ 3–5 lesions detected by the reference standard and no such lesion detected by the DL-CAD. TN was not defined at the lesion level [26].

### Interobserver agreement

Inter-observer agreement between the clinical reports and a reader group of 3 radiology trainees (Observer 2: M.B.; Observer 3: A.S.; and Observer 4: J.F.) for prostate lesion detection was performed in a subset of 98 patients using bp PI-RADS v2.1. Trainees were chosen for this analysis to evaluate how DL-CAD compares to this less experienced reader group in reproducing the observations of the reference standard.

### Statistical analysis

For per-patient assessment of lesion detection performance, sensitivity, specificity, PPV and NPV were obtained, as well as Cohen’s kappa for agreement between the DL-CAD and the reference standard. Receiver operator characteristic curves (ROCs) were also generated, and the corresponding areas under the curve (AUCs) with 95% confidence intervals for the detection of high-risk lesions (PI-RADS 3–5) by DL-CAD were obtained. For per-lesion analysis, sensitivity and PPV were calculated (as TNs were not defined). Lesion classification performance was assessed with a weighted kappa analysis restricted to PI-RADS ≥ 3 lesions identified by DL-CAD at the correct locations, as follows: poor agreement, < 0.20; fair agreement, 0.21–0.40; moderate agreement, 0.41–0.60; good agreement, 0.61–0.80; and excellent agreement, > 0.80.

Possible reasons for incorrect predictions by the DL-CAD were investigated by (1) independent review of FP and FN cases by 2 radiologists (Observer 1 and Observer 5, S.L., an expert radiologist with 13 years of experience), and (2) comparison of central tendencies/proportions of numeric/categorical clinical and imaging parameters—BMI, prostate volume, MR image quality, index lesion size and location—between patient groups of correct (TP + TN) and incorrect (FP + FN) DL-CAD predictions.

Descriptive statistics were used to summarize the clinical and imaging parameters of the study population. Intergroup comparisons for continuous variables were done using binary logistic regression to obtain odds ratios, whereas Chi-square and Fisher’s exact tests were used for categorical variables. Statistical significance was given by the condition *p* < 0.05. All statistical analyses were performed in R (R version 4.3.1).

## Results

### Reference standard

Among the 442 patients assessed, 274 PI-RADS ≥ 3 lesions (PI-RADS-3, *n* = 92; PI-RADS-4, *n* = 122; and PI-RADS-5, *n* = 60) were reported in 216 patients (1.27 lesions per patient) per the reference standard.

### MRI image quality results and interobserver agreement

The image quality scores were considered good-to-excellent in most cases (Table 1). The per-patient interobserver agreement between trainees and the reference standard was moderate-to-good for detection of PI-RADS ≥ 3/4/5 lesions (kappa = 0.66/0.53/0.71).

### Performance of DL-CAD in the detection of PI-RADS ≥ 3 lesions

The DL-CAD detected 285 PI-RADS ≥ 3 lesions (PI-RADS-3, *n* = 85; PI-RADS-4, *n* = 122; and PI-RADS-5, *n* = 78) in 205 patients (1.39 lesions per patient). 143/274 (52%) PI-RADS ≥ 3 lesions were detected at the same location by the DL-CAD and the reference standard.

#### Per-patient analysis

**Fig. 3:**
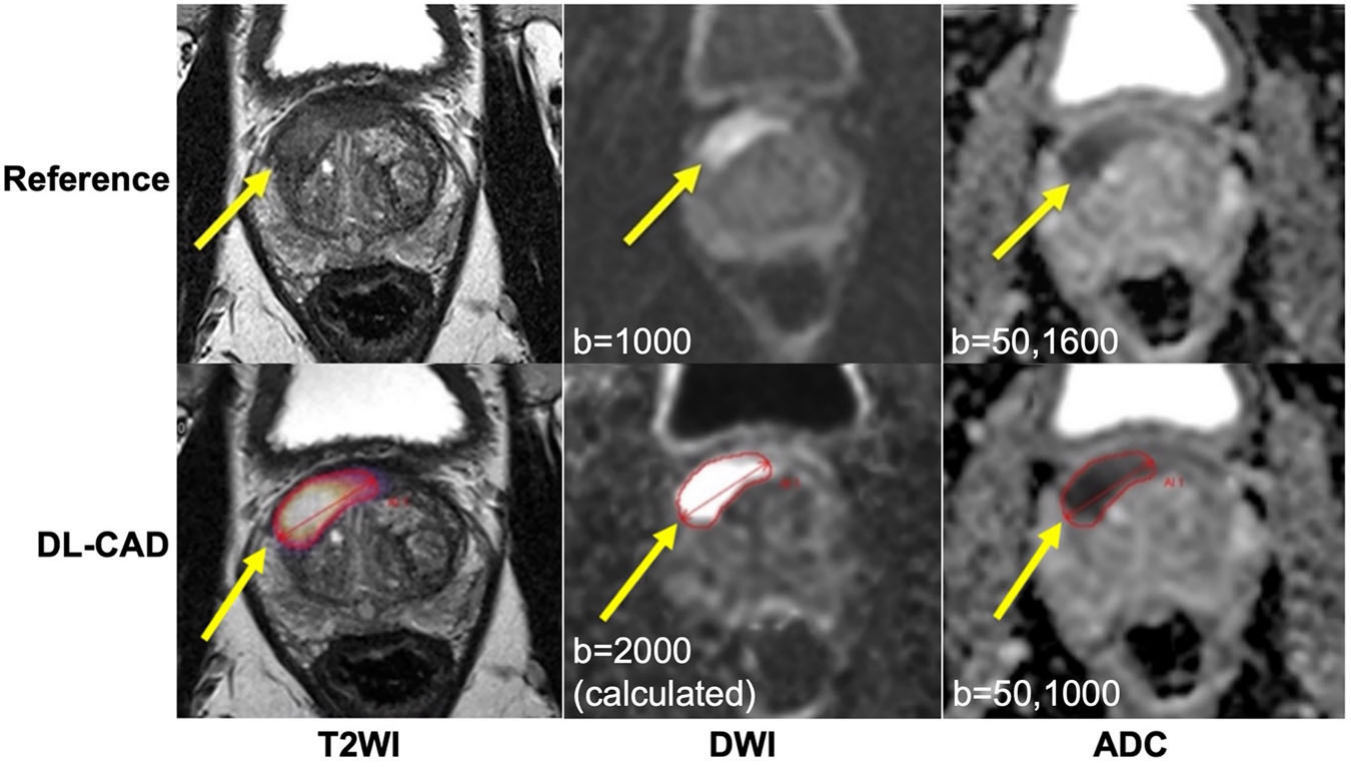
Agreement between deep learning-based computer-aided detection (DL-CAD) and the reference standard (clinical report). PI-RADS 5 lesion correctly identified in the right transitional zone in a 53-year-old man. ADC, apparent diffusion coefficient; DWI, diffusion-weighted imaging; T2WI, T2-weighted imaging

**Fig. 4:**
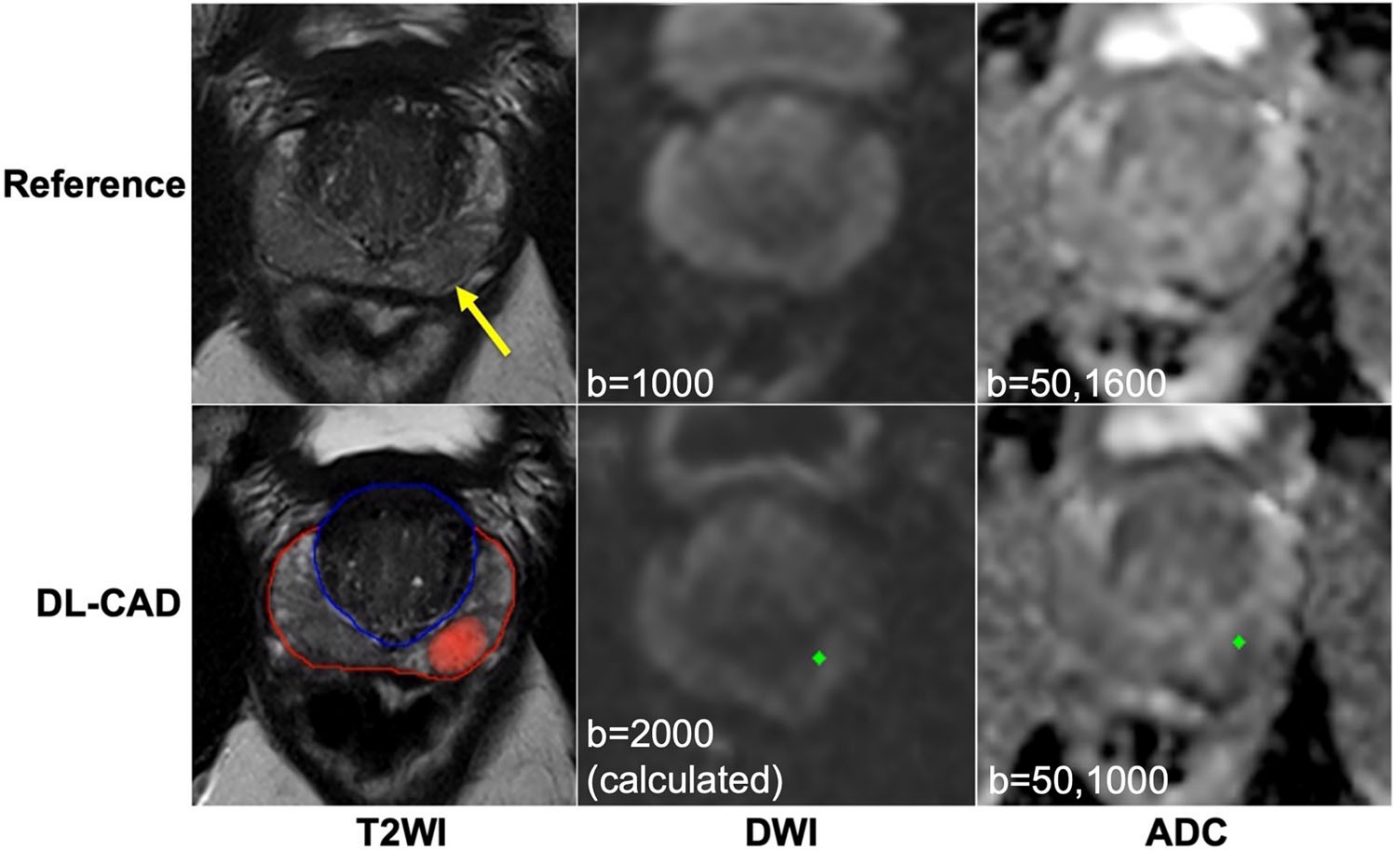
Example of false-positive detection using the deep learning-based computer-aided detection (DL-CAD) algorithm in a 54-year-old man. Lesion diagnosed as PI-RADS 4 by DL-CAD and PI-RADS 2 by the reference standard. ADC, apparent diffusion coefficient; DWI, diffusion-weighted imaging; T2WI, T2-weighted imaging

**Fig. 5:**
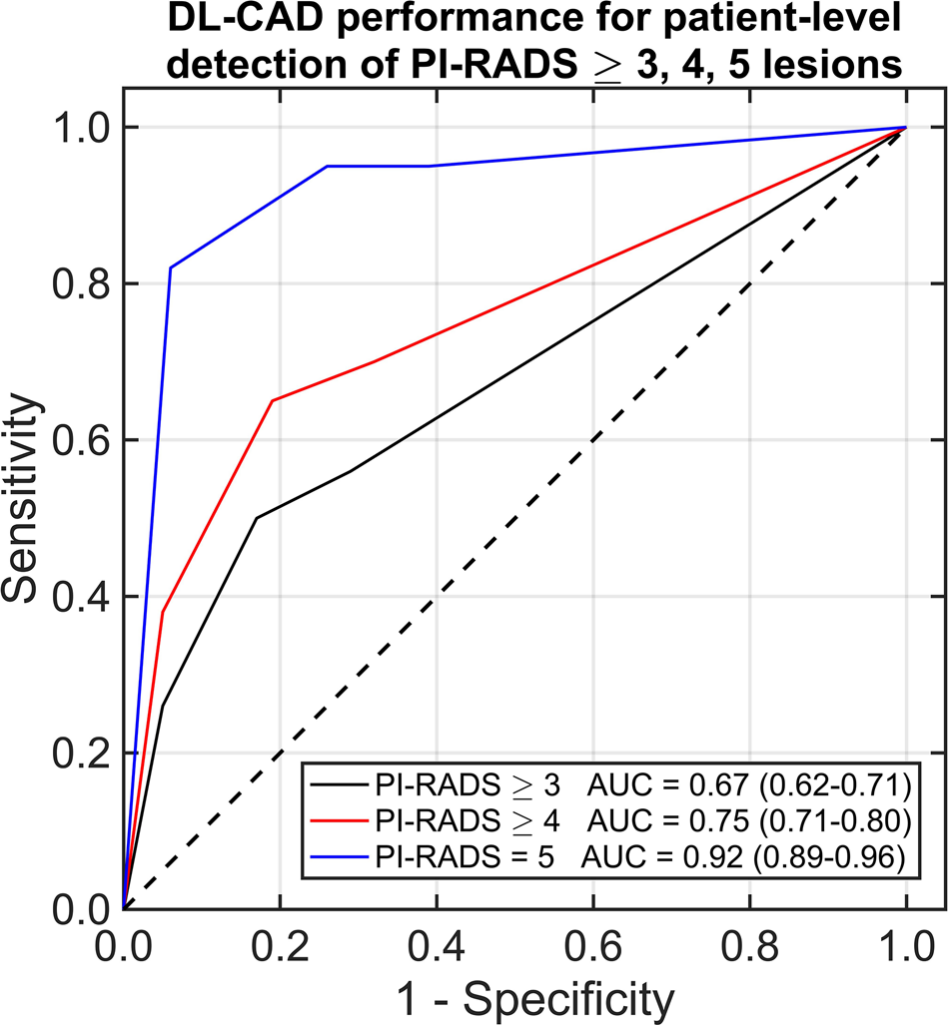
Receiver operator characteristic (ROC) curves for the detection of PI-RADS 3–5 lesions by the deep learning-based computer-aided detection (DL-CAD) algorithm relative to the reference standard in the per-patient analysis. Each curve represents the ROC for PI-RADS ≥ 3, 4, 5. AUC, area under the receiver operator characteristic curve

Agreement between DL-CAD and the reference standard for detection of PI-RADS 3–5/4–5/5 lesions was fair/moderate/good. Per-patient sensitivity, specificity, and diagnostic performance increased with higher PI-RADS scores (Table 2, Figs. 3–5). NPV was fair/good/excellent for PI-RADS ≥ 3/4/5 lesions (Table 2).

**Table 2:** Per-patient and per-lesion performance of DL-CAD for the detection of prostate lesions compared to the reference standard

	Patient-level performance		
	PI-RADS ≥ 3	PI-RADS ≥ 4	PI-RADS = 5
AUC	0.67 [0.62–0.71]	0.75 [0.71–0.80]	0.92 [0.89–0.96]
Sensitivity %	55.6 [48.9–62.2] (120/216)	65.3 [57.5–73.1] (94/144)	82.1 [72.1–92.2] (46/56)
Specificity %	70.8 [64.9–76.7] (160/226)	81.2 [76.8–85.6] (242/298)	93.8 [91.4–96.2] (362/386)
PPV %	64.5 [57.6–71.4] (120/186)	62.7 [54.9–70.4] (94/150)	65.7 [54.6–76.8] (46/70)
NPV %	62.5 [56.6–68.4] (160/256)	82.9 [78.6–87.2] (242/292)	97.3 [95.7–99.0] (362/372)
Kappa^a^	0.26 [0.18–0.35]	0.46 [0.37–0.55]	0.68 [0.59–0.78]

	Lesion-level performance		
	PI-RADS ≥ 3	PI-RADS ≥ 4	PI-RADS = 5
Sensitivity %	52.2 [46.3–58.0] (143/274)	60.4 [53.2–67.3] (110/182)	78.3 [66.4–86.9] (47/60)
PPV %	50.2 [44.4–55.9] (143/285)	55.0 [48.1–61.7] (110/200)	60.3 [49.2–70.4] (47/78)

95% confidence intervals are shown in square brackets, and total counts are shown in parentheses

*AUC* area under the receiver operator characteristic curve, *DL-CAD* deep learning-based computer-assisted diagnosis, *NPV* negative predictive value, *PPV* positive predictive value

^a^ Agreement with reference standard

#### Per-lesion analysis

Sensitivity was higher for PI-RADS 4–5 and 5 lesions. PPV showed less improvement as the PI-RADS cutoff was increased (Table 2).

### Performance of DL-CAD in the classification of PI-RADS ≥ 3 lesions

**Fig. 6:**
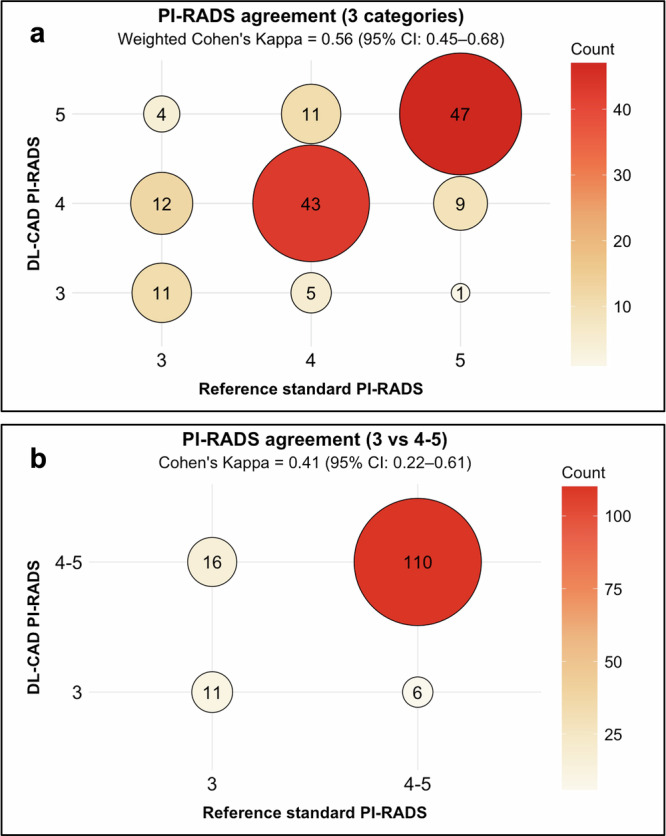
Performance of deep learning-based computer-aided detection (DL-CAD) algorithm in classification of high-risk lesions (PI-RADS 3–5) detected at concordant locations (lesion-based analysis). **a** Balloon plot for lesion classification as PI-RADS 3, 4, or 5. **b** Balloon plot for lesion classification as PI-RADS 3 vs 4–5. Numbers inside circles are the lesion counts

A concordance analysis for PI-RADS ≥ 3 lesions detected by DL-CAD and the reference at the same locations is shown in Fig. 6. Among the various incorrect predictions, we noted that DL-CAD overcalled 12 PI-RADS 3 lesions as PI-RADS 4, and 11 PI-RADS 4 lesions as PI-RADS 5. The resulting linearly weighted kappa value was moderate (kappa = 0.56; 95% CI: 0.45–0.68). Combining PI-RADS scores 4 and 5 into one category decreased kappa to 0.41 (CI: 0.22–0.61) (Fig. 6).

### Analysis of discordances between DL-CAD and the reference standard

Per-patient discordances in the detection of PI-RADS ≥ 3 lesions were retrospectively analyzed by two experienced radiologists (Observers 1 and 5). 33/66 (50%) of the FP cases were considered to represent benign prostatic hyperplasia (BPH) or possible scarring/changes of prostatitis. In 22/66 (33%) additional cases, the veru montanum, PI-RADS 2 lesions, and central zone insertions were also identified as suspicious lesions by the DL-CAD. In the 96 FN cases, 16 (17%) lesions were found to be located near the PZ-TZ junction, 7 (7%) in the PZ-periprostatic tissue, and 4 (4%) in the prostatic apex.

We investigated whether the source of discordances could be attributed to clinical (BMI) or imaging (prostate volume, lesion size and location, image quality) markers. For simplicity, the TP and TN detections were grouped into a category called “hits,” whereas the FP and FN detections were combined into a category called “misses.” This analysis showed that lesion size was the only significant factor for distinguishing DL-CAD hits from misses (OR = 3.67; 95% CI: 2.20–6.13; *p* < 0.001), with mean lesion sizes of 1.6 ± 1.0 cm and 1.0 ± 0.6 cm for detected and undetected lesions, respectively (Table 3). Other factors were not significant predictors of discordance.

**Table 3:** Performance of clinical and imaging markers in differentiating between DL-CAD hits (true positive + true negative cases) and misses (false positive + false negative cases)

Continuous predictors						
Predictor	Number of misses/hits	Misses^a^	Hits^a^	OR (95% CI)*		*p*-value*
BMI	150/261	27.3 ± 4.3	27.1 ± 4.2	0.99 (0.94–1.04)		0.703
Prostate volume (mL)	162/280	67.7 ± 37.6	66.7 ± 30.5	1.00 (1.00–1.00)		0.827
**Index lesion size (cm)**^b^	**99/125**	**1.0 ± 0.6**	**1.6 ± 1.0**	**3.67 (2.20–6.13)**		**< 0.001**
DWI image quality	162/280	4.6 ± 0.6	4.5 ± 0.6	0.85 (0.64–1.14)		0.288
T2WI image quality	162/280	4.5 ± 0.6	4.6 ± 0.7	1.15 (0.84–1.58)		0.387

Categorical predictors						
Predictor	Class	Misses	Hits	*p*-value (χ^2^)	*p*-value (Fisher)	
Index lesion location	PZ	62	82	0.512	0.477	
	TZ	37	39			

*BMI* body-mass index, *DWI* diffusion-weighted imaging, *OR* odds ratio, *PZ* peripheral zone, *T2WI* T2-weighted imaging, *TZ* transition zone

Statistically significant predictors are highlighted in bold

* Univariate logistic regression

^a^ Values reported are means ± SD

^b^ There were 224 patients with radiologist-reported lesions. Size measured on the reference standard

## Discussion

In our study, we assessed the performance of a pre-trained DL-CAD system for the detection and classification of prostate lesions in a real-world setting, including patients with benign and malignant findings. Per-patient and per-lesion analyses were performed to evaluate the performance of DL-CAD in lesion localization and classification.

The moderate-to-good interobserver agreement between radiology residents and the clinical reports supports our use of the PI-RADS scoring system as the reference. Using histopathology as the reference would have limited the analysis to biopsied patients, overrepresenting malignant lesions, which is not reflective of a real-world clinical setting.

AUC for per-patient detection of PI-RADS ≥ 3/4/5 lesions was fair-to-excellent, and agreement between DL-CAD and the reference standard was fair/moderate/good (per-patient analysis) for detection of PI-RADS ≥ 3/4/5 lesions, with improved performance for higher PI-RADS lesions. The DL-CAD also showed good/excellent performance in ruling out PI-RADS ≥ 4/5 lesions, as indicated by the high NPVs at the patient level. Per-patient agreement between DL-CAD and the reference standard was comparable to that between the trainee group and the reference standard in the detection of PI-RADS ≥ 4/5 lesions. However, the agreement between trainees and the reference standard was higher for PI-RADS ≥ 3 lesions.

To assess the performance of the DL-CAD system for PI-RADS classification, we focused on 143 lesions correctly detected by the DL-CAD system. The linearly weighted kappa was moderate for the classification of PI-RADS ≥ 3 lesions. However, this value was higher than that obtained in a similar assessment by Sanford et al [27] involving a semi-automated, convolutional neural network-based algorithm, although PI-RADS 2 lesions were also included in their analysis. In our cohort, there was a sizable fraction of these lesions where DL-CAD and radiologists assigned discordant PI-RADS = 3/4 scores. Combining PI-RADS scores 4 and 5 into one category skewed the distribution of PI-RADS 3 vs 4–5 lesions (27/143 vs 116/143), decreasing classification performance.

Using a cascaded DL-based algorithm trained on T2WI and DWI, Lin et al [12] reported per-patient sensitivity/specificity of 93%/23% for PI-RADS ≥ 2 lesions, which are considerably larger/smaller than our values. Our lower sensitivity may be attributed to our different per-patient TP counting, where TP detection required the correct localization of the respective index lesion. Lin et al also obtained a per-lesion sensitivity of 55% and a PPV of 57%, similar to our study. Our per-lesion analysis showed substantially lower sensitivities for PI-RADS ≥ 4 than those of attention-based and several U-Net-based CAD methods (77–93%) [13], presumably because those models were not tested in an external cohort. These comparisons showcase the variability in performance of proposed CAD algorithms found in the literature, which can come from differences in performance metrics, testing cohorts (internal vs external), overall lesion characteristics, or imaging protocols. A meta-analysis by Syer et al [28] showed a wide range of sensitivity and specificity when assessing various CAD algorithms in the ranges 47–97% and 41–91%, respectively, compared to histopathological reference.

In our study, we also assessed clinical and imaging factors that may affect the performance of DL-CAD, such as lesion size and location, prostate volume, BMI, and image quality. As expected, larger lesions were better detected by DL-CAD, as reported previously [29]. Performance for sub-centimeter lesions is limited by the large slice thickness of biparametric MRI, resulting in partial-volume effects that attenuate tumor-specific signal contrast and texture. In addition, small lesions are more susceptible to T2WI-DWI misregistration and often lack distinctive morphological features, which causes them to resemble benign lesions. Potential strategies to mitigate these limitations include enriching the training dataset with more lesions showing partial-volume effects, increasing representation of sub-centimeter lesions during training, using higher-resolution DWI at inference where available, and adopting multi-scale DL architectures that preserve fine spatial detail while capturing anatomical context.

Our image quality assessment showed insignificant differences in T2WI/DWI image quality between the hits (TP + TN) and misses (FP + FN) groups, presumably because of the high image quality for the entire cohort. Caglic et al [30] reported a drop in PPV of CAD with poor image quality, especially with rectal gas. We also found no correlation between the increased BMI of patients and DL-CAD performance.

Independent review of per-patient discordant detections revealed that, in 83% of the FP cases, BPH, scarring, and PI-RADS 2 lesions were mistaken for malignant lesions, whereas 28% of the lesions missed by DL-CAD (FN) were located at the PZ, the PZ-TZ junction, or the prostate apex. Even though lesion location accounts for a substantial portion of the FP detections, we found no significant difference in DL-CAD lesion detection rates between patients with lesions in PZ and TZ.

The high NPV of the DL-CAD for ruling out PI-RADS 4–5 lesions shows that patients classified as negative by the algorithm have a low risk of harboring clinically significant PCa, which could reduce unnecessary biopsies and their associated risks in a clinical setting. In contrast, the relatively low sensitivities observed suggest that the DL-CAD could benefit from additional training on more cases with BPH nodules, lesions located in the PZ-TZ junction, apex, and sub-cm lesions. This is especially relevant for lesion-based detection and classification, which could be potentially useful in disease staging and targeted biopsies, where accurate lesion localization is essential.

The main limitation of this study is the lack of histopathologic correlation. However, the aim of this study was to test how a DL detection and classification algorithm would perform in a clinical setting, and PI-RADS evaluations for prostate cancer risk are standard in clinical practice. Other limitations include the retrospective, single-center study design and its inherent selection bias, as well as the use of only two scanner manufacturers. Finally, no fine-tuning of the algorithm was performed to include populations representative of our institution. Further training of the DL-CAD in a more diverse cohort could improve its generalizability and diagnostic performance.

In conclusion, a DL-CAD showed good-to-excellent per-patient performance for the detection of PI-RADS ≥ 4 lesions and moderate performance of PI-RADS ≥ 3 lesion classification, with good-to-excellent performance in ruling out PI-RADS 4/5 lesions.

Knowledge of reasons for discordances between DL-CAD and clinical interpretation may aid in the refinement of the DL-CAD algorithm. Future prospective studies validating the DL algorithm with histopathologic correlation are warranted.

## Data Availability

Data generated or analyzed during the study are available from the corresponding author upon request.

## Abbreviations

ADC: Apparent diffusion coefficient
AUC: Area under the receiver operator characteristic curve
bpMRI: Biparametric MRI
CNN: Convolutional neural network
DL-CAD: Deep learning-based computer-aided detection
DWI: Diffusion-weighted imaging
PCa: Prostate cancer
PI-RADS: Prostate Imaging-Reporting and Data System
PZ: Peripheral zone
T2WI: T2-weighted imaging
TZ: Transitional zone
